# Melanotrichoblastoma misdiagnosed as basal cell carcinoma: a case report

**DOI:** 10.3389/fmed.2026.1725360

**Published:** 2026-01-26

**Authors:** Meng Zhang, Ruiqi Chu, Shengni Zhang, Chunmei Liu, Lihui Bian, Xiangxiang Ren

**Affiliations:** 1 Department of Dermatology, Affiliated Hospital of Hebei University, Baoding, China; 2 Department of Pathology, Affiliated Hospital of Hebei University, Baoding, China; 3 Department of General Surgery, Affiliated Hospital of Hebei University, Baoding, China

**Keywords:** basal cell carcinoma, diagnostic error, histopathology, melanotrichoblastoma, trichoblastom

## Abstract

**Background:**

Melanotrichoblastoma (MTB) is an exceptionally rare benign adnexal tumor with follicular differentiation. Its clinical and dermoscopic resemblance to pigmented basal cell carcinoma (BCC) often leads to misdiagnosis.

**Case presentation:**

A 51-year-old male presented with a slow-growing, blue-black nodule on the right canthus that had been present for five decades, with recent central ulceration. Dermoscopy revealed homogeneous pigmentation with atypical blue-white areas and dilated vessels, suggestive of BCC. Histopathological examination showed well-demarcated dermal nests of basaloid cells with peripheral palisading and stromal pigment deposition. Importantly, retraction clefts were absent, a key feature distinguishing MTB from BCC. The lesion was completely excised, with no recurrence after two years.

**Conclusion:**

This case highlights the diagnostic challenge of MTB. Histopathology remains the gold standard for differentiation, wherein the absence of retraction clefts serves as a pivotal diagnostic clue. Increased awareness of MTB is essential to avoid misdiagnosis and ensure appropriate, conservative management.

## Introduction

Cutaneous adnexal tumors comprise a diverse group of neoplasms with differentiation toward skin appendages. Among them, trichoblastoma (TB) is a rare, benign tumor thought to originate from follicular germinative cells. Melanotrichoblastoma (MTB) is an exceptionally uncommon pigmented variant. It typically presents as a slow-growing, firm nodule on the head or neck of middle-aged to older adults, though its clinical appearance is often non-specific. MTB poses a significant diagnostic challenge because its dark coloration and dermoscopic features frequently mimic the far more prevalent pigmented basal cell carcinoma (BCC) (1, 2). This overlap often leads to misdiagnosis. Accurate distinction is clinically paramount, given their differing biological behavior and management. BCC, a malignant neoplasm, may require extensive surgery with margin assessment, whereas MTB is benign and cured by simple, complete local excision. Histopathological examination is the definitive diagnostic tool, with key discriminators including the architectural pattern of basaloid cells, the presence or absence of stromal retraction clefts, and immunohistochemical profiles. This case report of an MTB initially misdiagnosed as BCC underscores the importance of considering this rare entity in the differential diagnosis of pigmented skin tumors.

## Case presentation

A 51-year-old male presented to our outpatient department with a long-standing skin lesion on the posterior aspect of the right canthus. The lesion, present for 50 years, had recently ulcerated. The patient reported that a millet-sized black papule had appeared during childhood and had gradually enlarged over decades without treatment. In recent months, central ulceration developed without significant pain or pruritus. His past medical and family history were unremarkable.

**Figure 1:**
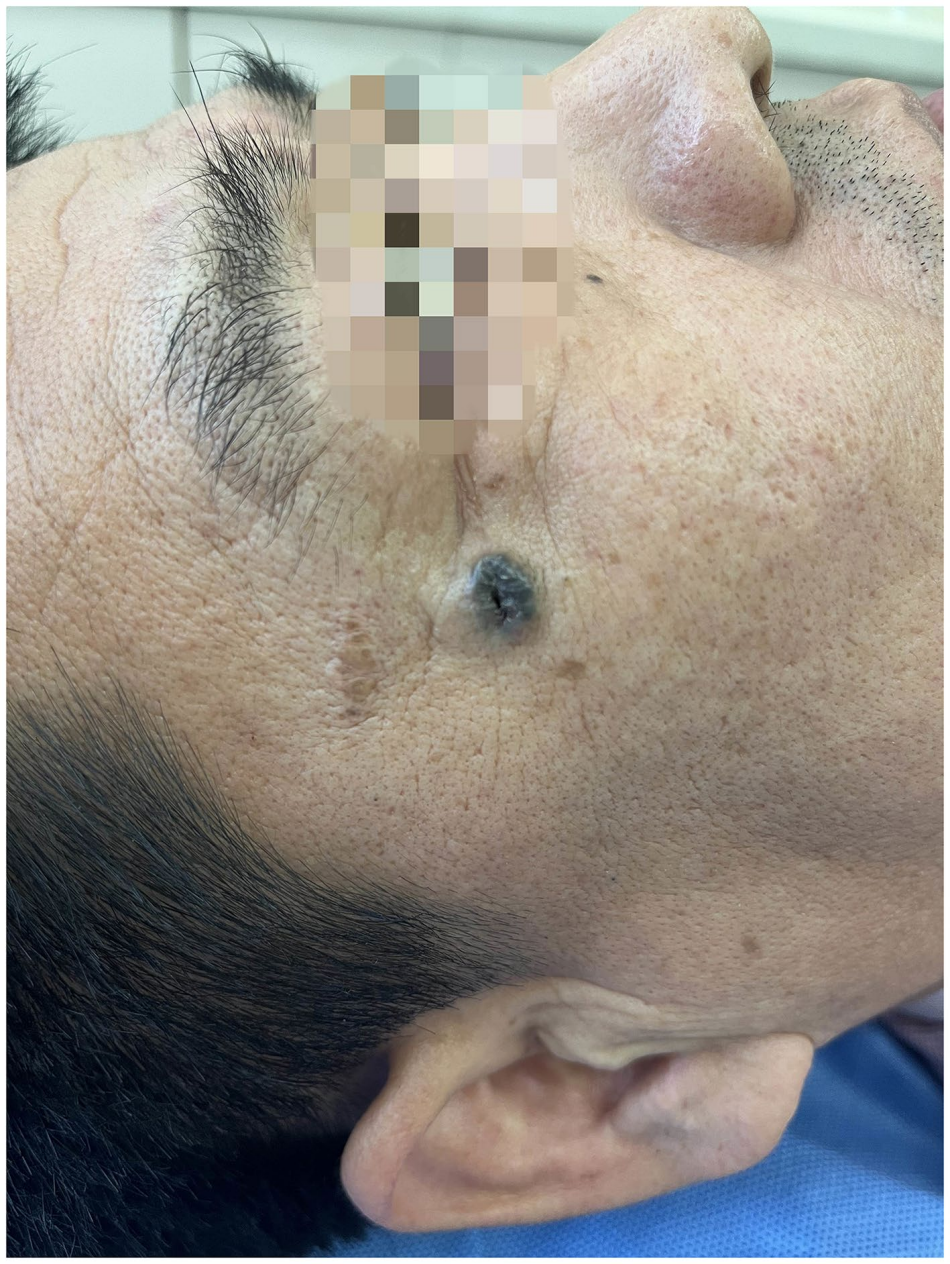
Clinical presentation of the lesion. A well-circumscribed, firm, blue-black nodule located posterior to the right canthus. Note the central ulceration and darker coloration in the center compared to the edges.

**Figure 2:**
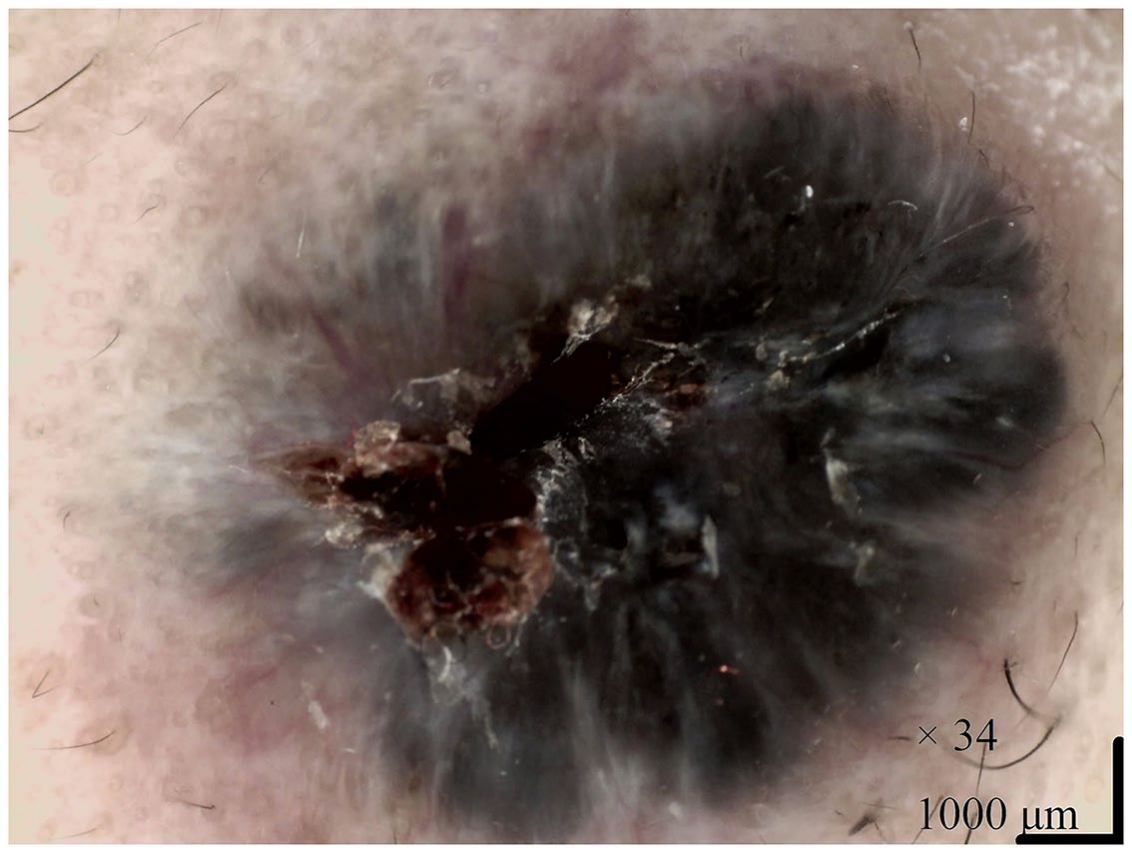
Dermoscopic examination of the lesion. The image shows a symmetrical, black raised structure with relatively regular borders. Key features include homogeneous pigmentation, atypical blue-white structures, a central blood crust, and surrounding dilated vessels.

Physical examination revealed the patient to be in good general condition. Dermatological examination showed a well-defined, firm, blue-black nodule, approximately 1.0 cm × 1.0 cm in size, located posterior to the right canthus. The center was darker and exhibited a depressed ulceration (Figure 1). The nodule was mobile and non-tender. Dermoscopy revealed a largely symmetrical, black raised structure with relatively regular borders. It displayed homogeneous pigmentation with atypical blue-white structures, a central blood crust, and surrounding dilated vessels (Figure 2). We made a preliminary diagnosis of “Basal Cell Carcinoma?” and performed a skin biopsy.

**Figure 3:**
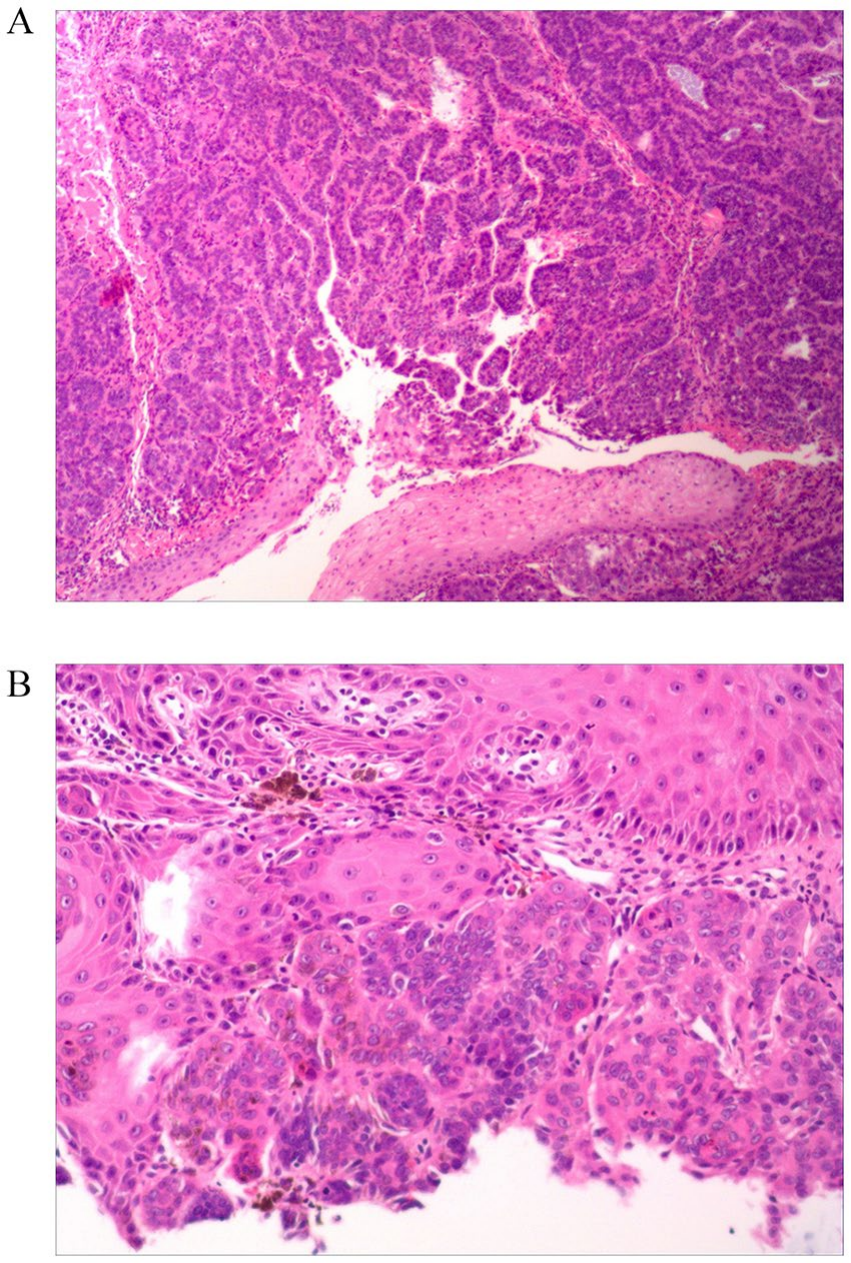
Histopathological findings (hematoxylin and eosin staining). **(A)** Low-power view (40x) showing well-demarcated nests and cords of basaloid cells within the dermis, separated by fibrous stroma. The epidermis is uninvolved. **(B)** High-power view (200x) of a tumor nest demonstrating basaloid cells with peripheral palisading and abundant stromal pigment deposition. Critically, there is an absence of retraction clefts between the tumor nests and the surrounding stroma.

Histopathological examination revealed well-defined nests and cords of basaloid cells within the dermis. The cells showed no significant atypia and had oval or cuboidal shapes with basophilic nuclei. Peripheral palisading was observed at the nests’ periphery. The tumor nests were separated by collagen fibers with prominent pigment deposition. Notably, retraction clefts between the tumor nests and the surrounding stroma were absent (Figure 3). These histopathological findings, particularly the lack of retraction clefts, were inconsistent with the initial clinical and dermoscopic impression of BCC and confirmed the diagnosis of melanotrichoblastoma. The lesion was completely excised via superficial mass resection. The postoperative wound healed well, and no recurrence was observed during a two-year follow-up period.

## Discussion

We report a case of melanotrichoblastoma initially misdiagnosed as pigmented basal cell carcinoma due to its prolonged history, ulcerated nature, and suspicious dermoscopic features. This case epitomizes a well-recognized diagnostic challenge in dermatology and dermatopathology. Although MTB is a benign tumor, its clinical and dermoscopic presentation can strikingly mimic the more common and malignant BCC (1, 3). To the best of our knowledge, only seven cases of MTB have been previously reported in the English literature (Table 1), making our case the eighth documented instance and further underscoring its rarity.

**Table 1:** Summary of reported cases of melanotrichoblastoma (including the present case).

Case No.	Reference (Source document)	Age (years)	Sex	Tumor location	Size (cm)	Associated with Nevus Sebaceus?	Key characteristics
1	Kanitakis et al. (4)	32	Female	Scalp	2.0	No	First reported case; heavy melanin deposits; dendritic melanocytes (S100+, tyrosinase+, MART-1/HMB45+); clinically mimicking blue nevus or melanoma
2	Kim et al. (5)	51	Male	Back	6.0	No	Giant subcutaneous MTB; Melan-A+, HMB45+, S-100+; differential diagnosis from BCC emphasized
3	Hung et al. (6)	31	Male	Scalp	1.0	Yes	Ripple-pattern MTB within nevus sebaceus; HMB45 + dendritic melanocytes; blue-black nodule
4	Mizuta et al. (7)	72	Female	Right lower leg	Approx. 11.0	No (collision with SK)	Collision tumor with seborrheic keratosis; dermoscopy: blue-gray ovoid nest + white structures; no recurrence at 26 months
5	Ocanha-Xavier et al. (1)	59	Female	Scalp	0.8	No	Sixth formally reported case; basaloid nests with pigment; CK AE1/AE3+, Melan-A+; asymptomatic
6	Stoyanov et al. (3)	51	Female	Mons pubis	2.0	No	First reported on pelvic girdle; clinically resembled ingrown hair; IHC: CK AE1/AE3+, BerEP4+, Melan-A+, HMB45+
7	Thekho et al. (8)	33	Female	Scalp	4.0	Yes	Arising in nevus sebaceus; slow-growing pigmented tumor; IHC: S100+, HMB45 + dendritic melanocytes
8	Present case	51	Male	Right canthus	Approx. 1.0	No	Ulcerated blue-black nodule; dermoscopy: blue-white structures + vessels; histology: basaloid nests with pigment, NO retraction clefts; confirmed MTB; no recurrence at 24 months

In addition to pigmented basal cell carcinoma, the preoperative differential diagnosis included melanoma, pigmented trichoblastoma, pigmented seborrheic keratosis, and vascular lesions such as traumatic angiokeratoma. Although the lesion appeared blue-black with ulceration clinically, melanoma was considered. However, it was not the primary diagnostic consideration based on the following: dermoscopy revealed none of the typical structures of melanoma (such as an irregular pigment network, pseudopods, or radial streaming), and the lesion demonstrated an extremely slow growth pattern over 50 years—a clinical course more suggestive of a benign or low-grade malignant process. Nevertheless, melanoma should always be considered in the differential diagnosis of any pigmented nodule, with definitive diagnosis relying on histopathological examination. Traumatic angiokeratoma often presents as a blue-black papule or nodule, typically located in trauma-prone areas such as the extremities, and may appear dark due to hemorrhage. Dermoscopy usually reveals red to blue-black lacunar structures, which differ from the homogeneous pigmentation and vascular patterns observed here. The final histopathological diagnosis was melanotrichoblastoma. Key distinguishing features from other conditions include the presence of stromal retraction clefts, degree of cellular atypia, and the immunohistochemical expression profile.

The diagnostic dilemma in our patient was compounded by ulceration, an uncommon feature in TB/MTB that typically raises suspicion for malignancy (4–9). Furthermore, the dermoscopic findings—including homogeneous pigmentation, atypical blue-white structures, and dilated vessels (Figure 2)—are highly characteristic of pigmented BCC (5, 10). This aligns with previous reports describing non-specific, BCC-like dermoscopic patterns in MTB, where features such as blue-gray ovoid nests and arborizing vessels can also be observed (1, 6, 11). In contrast, classic benign trichoblastomas more frequently display a white background and fine, short telangiectasias (2). The overlap in dermoscopic features between these entities necessitates a low threshold for histopathological confirmation. Additionally, in patients with darker skin tones, the dermoscopic features of pigmented basal cell carcinoma often present as ovoid nests and black dots, which were not observed here, further supporting differentiation from typical pigmented BCC. Literature suggests that dermoscopic presentations of adnexal tumors in patients with skin of color may vary, necessitating comprehensive analysis in conjunction with histopathology (12). Ultraviolet dermoscopy may offer greater diagnostic value in such patients. Although not utilized in this case, future studies may consider incorporating it to enhance diagnostic accuracy.

Histopathological examination remains the unequivocal gold standard for differentiating MTB from BCC. Our case exhibited hallmark features of MTB: well-demarcated nests and cords of basaloid cells within the dermis, demonstrating peripheral palisading and set in a fibrous stroma with prominent melanin deposition. Several critical histological distinctions secure the diagnosis: Architectural and Stromal Relationship: MTB typically presents as a well-circumscribed dermal tumor without connection to the epidermis. The most pivotal differentiating feature is the absence of retraction clefts between the tumor cell nests and the surrounding stroma (13), a finding conspicuously present in our case (Figure 3B). In contrast, BCC frequently exhibits epidermal connection and characteristic stromal retraction artifacts. Cytological Features: The basaloid cells in MTB typically lack significant nuclear atypia or mitotic activity, helping distinguish it from more aggressive basal cell carcinomas. Immunohistochemical Profile: While not always necessary, immunohistochemistry can provide decisive evidence. The immunophenotype of MTB often shows patchy or peripheral staining for Bcl-2, CD10 positivity predominantly in the peritumoral stroma, and general negativity for Androgen Receptor (AR) (14, 15). This profile contrasts with BCC, which typically shows diffuse Bcl-2 positivity within tumor islands, CD10 positivity in the tumor cells themselves, and frequent AR positivity (14, 15).

In line with previously reported cases (Table 1), our patient demonstrates typical features of MTB, such as a pigmented nodular presentation on the head and neck, occurrence in middle age, and the clear absence of stromal retraction clefts on histopathology. However, ulceration introduces an unusual clinical characteristic, further highlighting the potential for misdiagnosis. Most reported cases present as pigmented nodules on the head and neck, with colors ranging from light brown to dark blue-black. Ulceration is relatively uncommon; its occurrence here may be related to the lesion’s prolonged duration and local friction. Although MTB has been documented in association with nevus sebaceus (6, 8), the vast majority of cases are sporadic, as in this instance.

As a benign neoplasm with negligible malignant potential, MTB is managed conservatively. Complete surgical excision is the treatment of choice and is curative. Long-term follow-up in our patient and in other reported cases (1, 7) confirms an excellent prognosis with no recurrence, provided the lesion is entirely removed.

## Conclusion

This case underscores the critical importance of considering melanotrichoblastoma in the differential diagnosis of pigmented nodular lesions, particularly on the head and neck. The significant clinical and dermoscopic overlap with pigmented basal cell carcinoma can readily lead to misdiagnosis and potential overtreatment. Histopathology is indispensable for definitive diagnosis, with key discriminators being well-defined dermal nests of basaloid cells, abundant stromal pigment, and, most critically, the absence of retraction clefts. Increased awareness of this rare entity among clinicians and pathologists is essential to ensure accurate diagnosis, guide appropriate conservative surgical management, and prevent unnecessary extensive procedures.

## Data Availability

The original contributions presented in the study are included in the article/supplementary material, further inquiries can be directed to the corresponding author/s.
